# Crop Phenology Detection Using High Spatio-Temporal Resolution Data Fused from SPOT5 and MODIS Products

**DOI:** 10.3390/s16122099

**Published:** 2016-12-10

**Authors:** Yang Zheng, Bingfang Wu, Miao Zhang, Hongwei Zeng

**Affiliations:** 1 Key Laboratory of Digital Earth, Institute of Remote Sensing and Digital Earth, Chinese Academy of Sciences, Beijing 100101, China; zhengyang@radi.ac.cn (Y.Z.); zhangmiao@radi.ac.cn (M.Z.); zenghw@radi.ac.cn (H.Z.); 2 College of Resources and Environment, University of Chinese Academy of Sciences, Beijing 100049, China

**Keywords:** SPOT5, MODIS, STARFM, data fusion, phenology detection

## Abstract

Timely and efficient monitoring of crop phenology at a high spatial resolution are crucial for the precise and effective management of agriculture. Recently, satellite-derived vegetation indices (VIs), such as the Normalized Difference Vegetation Index (NDVI), have been widely used for the phenology detection of terrestrial ecosystems. In this paper, a framework is proposed to detect crop phenology using high spatio-temporal resolution data fused from Systeme Probatoire d'Observation de la Tarre5 (SPOT5) and Moderate Resolution Imaging Spectroradiometer (MODIS) images. The framework consists of a data fusion method to produce a synthetic NDVI dataset at SPOT5’s spatial resolution and at MODIS’s temporal resolution and a phenology extraction algorithm based on NDVI time-series analysis. The feasibility of our phenology detection approach was evaluated at the county scale in Shandong Province, China. The results show that (1) the Spatial and Temporal Adaptive Reflectance Fusion Model (STARFM) algorithm can accurately blend SPOT5 and MODIS NDVI, with an *R*^2^ of greater than 0.69 and an root mean square error (RMSE) of less than 0.11 between the predicted and referenced data; and that (2) the estimated phenology parameters, such as the start and end of season (SOS and EOS), were closely correlated with the field-observed data with an *R*^2^ of the SOS ranging from 0.68 to 0.86 and with an *R*^2^ of the EOS ranging from 0.72 to 0.79. Our research provides a reliable approach for crop phenology mapping in areas with high fragmented farmland, which is meaningful for the implementation of precision agriculture.

## 1. Introduction

The phenology dynamics of regional vegetation reflect how ecosystems are responding to climate change, and the timing of phenological cycles is often used as an effective parameter for gaining a better understanding of vegetation-climate interactions and their implications on carbon cycling [1,2,3]. In the case of crops, phenology provides crucial information for irrigation scheduling, fertilizer management, seasonal ecosystem carbon dioxide (CO_2_) exchange cognition, and biomass productivity estimation [4,5]. Therefore, timely and accurate crop phenology detection is not only essential for climate variability research but also significant for the scientific management and rational utilization of farmland.

Traditionally, most crop phenology identifications involve time-consuming and laborious field surveys. In recent years, with the development of remote sensing technology, a growing number of studies have focused on utilizing satellite data to detect the phenology of different crops because the frequent remotely sensed images have significant potential for monitoring vegetation dynamics [6,7]. At present, to the best of the authors’ knowledge, phenology detections are mainly available at medium or coarse levels because high-spatial-resolution images are constrained by either low temporal resolution or low repeat cycles, and thus, it is almost impossible for these data to capture rapidly changing crop phenology. Meanwhile, coarse spatial resolution data, such as the Moderate Resolution Imaging Spectroradiometer (MODIS) [5,6,8], Systeme Probatoire d’Observation de la Tarre-Vegetation (SPOT-VGT) [9,10], and NOAA Advanced Very High Resolution Radiometer (AVHRR) images [11,12], are not suitable for phenology mapping in areas with fragmented landscapes because mixed pixels may seriously affect the spectral characteristics in the coarse satellite images [8,13]. In some parts of China, this limitation is more obvious because farmland is owned by different individuals and is generally divided into small portions [14]. Therefore, detecting crop phenology at a high spatial resolution is essential and extremely urgent in regions with small-scale fields.

Currently, although advances in satellite remote sensing provide more data source choices, data with both high spatial resolution and high temporal resolution for extracting crop phenology at a regional scale remain unavailable. To generate high spatial and temporal resolution data, various fusion algorithms have been proposed and proven practicable. Gao et al. developed a Spatial and Temporal Adaptive Reflectance Fusion Model (STARFM) algorithm for blending Landsat ETM+ and MODIS data to generate daily surface reflectance data at ETM+ spatial resolutions firstly. Subsequently, several modified methods aiming at improving the performance of the STARFM algorithm were presented [15]. For example, the Spatial Temporal Adaptive Algorithm for mapping Reflectance Change (STAARCH) method, which was developed by Hilker et al., allows for an optimal input Landsat image to be chosen and hence improves the accuracy of synthetic data [16]. Zhu et al. proposed an Enhanced Spatial and Temporal Adaptive Reflectance Fusion Model (ESTARFM) and obtained more accurately blended land surface reflectance data, especially over complex heterogeneous landscapes [17]. Recently, both STARFM and ESTARFM have been successfully applied for the fusion of Landsat and MODIS data in different environments [18,19,20,21], and several studies have attempted to test the feasibility of these algorithms to fuse MODIS data with high-spatial-resolution data from other space-borne sensors [22], which is very important for agriculture monitoring on more sophisticated levels. For example, Liu et al. derived phenological parameters of C3 and C4 vegetation types at high spatial resolution through fusing Landsat and time-series MODIS products [23]. Singha et al. have extracted the phenology of rice in India based on the high spatio-temporal resolution data blended from China’s Environmental Satellite (HJ-1A/B) and MODIS data [24]. All these applications have demonstrated the potential of data fusion methods in crop phenology detection.

The objective of the presented study is to propose a framework for crop phenology detection using high spatio-temporal resolution data blended from Systeme Probatoire d'Observation de la Tarre5 (SPOT5) and MODIS products in spatially heterogeneous landscapes. In this research, we want to answer two questions: (1) Is the STARFM algorithm appropriate for generating time-series data with multiple combinations of SPOT5 and MODIS data sets; (2) Is the time-series data combining SPOT5- with STARFM-predicted images suitable for detecting crop phenology changes in a typical region with high fragmented farmland in the North China Plain (NCP)? 

## 2. Study Area and Data

### 2.1. Study Area

**Figure 1:**
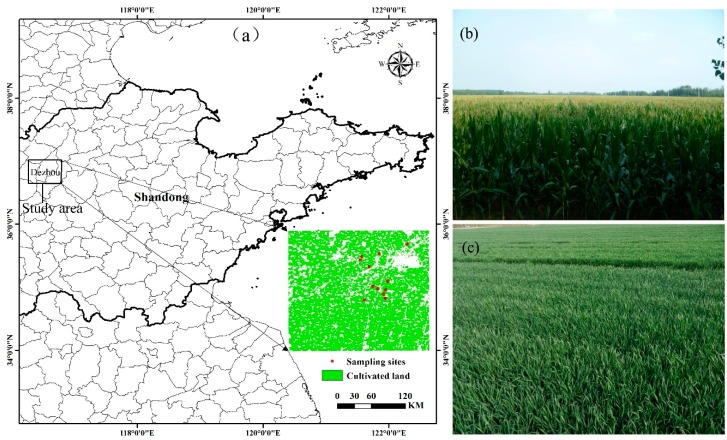
Location and photographs of the study site. (**a**) Location of study area and sampling sites; (**b**) Photograph of summer maize on 12 August 2015; (**c**) Photograph of winter wheat on 17 April 2015.

The study was conducted in Dezhou city (115°45′–117°36′ E, 36°24′–38°1′ N), which is situated in the western Shandong Province, one of the major production zones in China (as shown in Figure 1). This area is dominated by irrigated agriculture and moist soil, with an annual mean temperature and precipitation of 13.3 °C and 555.5 mm, respectively, concentrated from July to September. The farming structure of the study site has typical characteristics of the planting pattern of the NCP, mainly a rotation of winter wheat and summer maize, with occasional cotton and cash crops. The winter wheat is regularly sowed in mid-October and harvested in early June (wheat season), whereas summer maize is planted in mid-June and harvested in late September (maize season).

### 2.2. Data Acquisition and Processing

#### 2.2.1. Ground Data

Field observation data of the crop phenology calendar from 2014 to 2015 within the study region, such as the SOS and EOS, were collected from 18 and 15 fields (sampling sites) during the growing season of winter wheat and summer maize to validate the phenology estimations. In particular, only relatively homogeneous and large fields which were comparable with the 250-m pixels of MODIS data were selected. A handheld global positioning system (GPS) from Unistrong Science & Technology Co., Ltd. (Beijing, China), with a positional accuracy of <5 m was used to record the location of each field [25,26]. Once the observation positions were determined, we went to the study area every month to survey the phenology information. But for the artificially planted crops, monthly frequency may not be sufficient because crops change quickly, so we also asked several local farmers to help us, they can record the phenological features (as shown in Table 1) every 5–10 days and thus can make up for our low observation frequency, the specific phenological date of each field was defined as when more than fifty percentage crops in this field has reached the same phenology stage.

In particular, the SOS and EOS of winter wheat are corresponding to the jointing and maturity stages, while the SOS and EOS of summer maize are corresponding to the seven leaf and maturity stages of the ground observations. During the investigation periods, the crop and land use types were also recorded for the classification of different crops.

#### 2.2.2. Satellite Data

Generally, daily and 8-day maximum value compositing (MVC) reflectance data can be obtained from MODIS products. Although the daily data can better capture phenological differences, we selected the 8-day composite MOD09Q1 product because the daily data can be easily affected by many unavoidable factors such as clouds and haze. The MOD09Q1 data consist of red (R) and near-infrared (NIR) bands, and the spatial resolution is 250 m. The title number of the MODIS data which covered the study area is H27V05 and the MODIS product used in this study were from DOY281 (281st day of the year) in 2014 to DOY289 (289th day of the year) in 2015, a period covered the entire growing period of wheat and maize.

The SPOT5 satellite was successfully launched in May 2002 and can obtain high-spatial-resolution data (10 m in the visible and near-infrared region and 20 m in the shortwave infrared region), with a four-band spectrum ranging from 0.49 to 0.68 μm in the visible region (green and red bands) and from 0.78 to 1.78 μm in the infrared region (near-infrared and shortwave infrared bands). In total, we obtained ten SPOT5 images with less than 10% cloud cover during the 2014–2015 agricultural year, as shown in Table 2. Moreover, the study area has a sample size of 3988 × 4240 pixels.

#### 2.2.3. Auxiliary Data

An arable land mask derived from ChinaCover 2010 [27] and all the available multi-temporal SPOT5 images which were also used for data fusion were employed to generate spatial distributions of wheat and maize in 2015 based on the support vector machine (SVM) method. The phenology maps were then produced over the crop planting regions.

## 3. Methods

### 3.1. Data Fusion

Recently, although many data fusion methods developed based on the STARFM algorithm have been proven to improve the fusion accuracy in relevant research, STARFM was selected as the algorithm to generate high-spatial-resolution time-series data because it is the foundation of other fusion methods and because it has fewer restrictions. We used STARFM to directly blend vegetation indices (VIs) derived from SPOT5 and MOD09Q1 because several studies have found that STARFM method performs better when directly fusing VIs rather than when the reflectance is fused and then the VIs are calculated [28,29]. The NDVI (Normalized Difference Vegetation Index) (Equation (1)) was chosen because it has been proven to be an effective indicator for phenology extraction [30]:

(1) $$\mathrm{NDVI}=\left(\rho_{NIR}-\rho_{R}\right)/\left(\rho_{NIR}+\rho_{R}\right)$$

where $\rho_{NIR}$ and $\rho_{R}$ represent the land-surface reflectance of the near-infrared and red bands, respectively.

The STARFM algorithm requires at least one pair of high- and coarse-resolution images that were obtained in the same period and a series of coarse resolution images for the desired dates to predict high-spatial-resolution data. The implementation of STARFM was divided into two parts (wheat season and maize season) because large variations in land surface may occur over different seasons. Specifically, when taking growing stages into consideration, the SPOT5 data on 23 April (DOY113) and the MOD09Q1 product on DOY113 (represents a maximum value composite data over a 8 day period from DOY113 to DOY120) were selected as one pair, and the SPOT5 data on 16 August (DOY228) and the MOD09Q1 product on DOY225 were chosen as another pair to predict the high spatial and temporal resolution NDVI, therein covering the whole growth period of wheat and maize, respectively. The remaining SPOT5 images were used to evaluate the accuracies of the blended NDVI. Prior to implementing the STARFM data fusion algorithm, we used the MODIS Reprojection Tools (MRT) to reproject and resample the MODIS data to the projection and spatial resolution of the SPOT5 image. There is no need to further process the SPOT5 data because they were provided pre-processed for geometric and atmospheric corrections by THEIA land data center [31].

### 3.2. Data Smoothing

**Figure 2:**
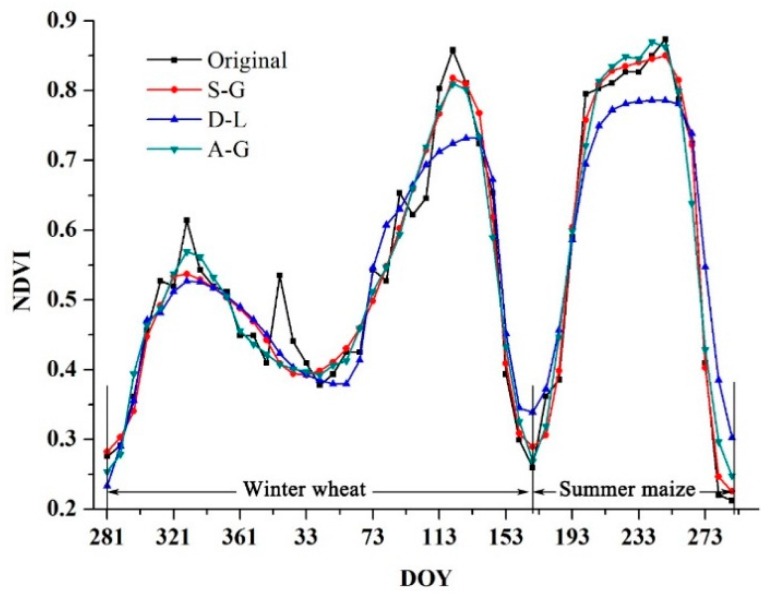
The original and filtered time-series NDVI profiles.

The averaged time-series NDVI profile of the study area for wheat and maize which extracted from MODIS data over the 2014–2015 agricultural season is shown in Figure 2, from which the phenomenon can be obtained that there were still fluctuations in the original time-series NDVI profile even though the reflectance had been composited, which was most likely caused by climate and atmospheric changes.

Because undesirable noises may influence the accuracy of the phenology extraction results, it is essential to eliminate such noises. In past decades, various filtering algorithms have been proposed and employed to reconstruct time-series data. However, an affirmatory method that could always achieve optimal results still did not exist. In this research, three different filtering methods, asymmetric Gaussian functions (A-G), double logistic functions (D-L) and Savitzky-Golay (S-G) filtering in the TIMESAT software, were tested to smooth the time-series NDVI. As shown in Figure 2, the S-G filtering method performed better for noise removal than did the Gaussian and logistic functions even though noise remained in the profile, which agreed with several previous studies [32]. Therefore, S-G filtering was chosen to smooth the time-series data. In addition, the periods for winter wheat and summer maize (i.e., the dash line in the figure) were determined by the observation dates shown in Table 1, and there appears to be two peaks in the NDVI profiles during the wheat season, as wheat entered a dormant period, during which the NDVI decreased from the first peak due to low temperatures and wheat almost stopped to grow, while the respiration is still normal, therefore the actual greenness was going down [33], then the NDVI moved towards the second peak. In this study, we only distinguished the SOS of wheat when it recovered from dormancy.

### 3.3. Phenology Detection

Among the numerous methods for deriving seasonal parameters from the time-series NDVI, the threshold method, which assumes that a specific phenology will start if the NDVI value exceeds a previously defined threshold, is widely applied because it generally keeps dates within a certain reasonable range and can thus achieve relatively high accuracies [9,34]. In general, the setting of thresholds is usually based on the characteristics of the NDVI curve; however, these characteristics vary with crop type changes, and different crops have their own phenological stages during their growing seasons [35]. In this study, we adopted the method developed by Pan et al. based on the ratio of NDVI*_min_* and NDVI*_max_* (NDVI*_ratio_*) over a specific period for crops to determine thresholds of the SOS and the EOS [14]. Here, specific periods were defined as from the beginning to the peak and from the peak to the end of the season to identify the NDVI*_min_* for the definitions of the SOS and the EOS. Since the NDVI*_min_* may be different at the start and end of season, the threshold values are also varied for the SOS and the EOS. The NDVI ratio was defined as follows:

(2) $${\mathrm{NDVI}}_{ratio}={\mathrm{NDVI}}_{min}/{\mathrm{NDVI}}_{max}$$

where NDVI*_min_* and NDVI*_max_* are the annual minimum and maximum values of a specific range of time-series NDVI, respectively.

**Figure 3:**
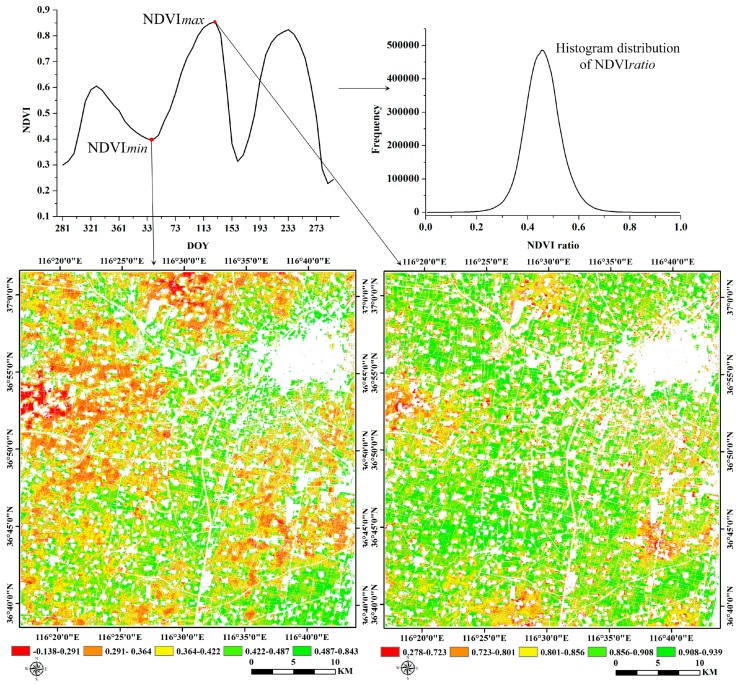
The calculation of NDVI*_ratio_* of SOS for winter wheat and threshold value were then determined based on the highest probability of NDVI*_ratio_*.

The threshold value for the SOS and EOS was dependent on the highest probability of the NDVI ratio, Figure 3 provides an example of the NDVI ratio and threshold calculation of SOS for winter wheat. In our study, the NDVI ratio of the SOS and EOS for wheat was calculated by NDVI*_min_* on day 41 and NDVI*_max_* on day 129, and NDVI*_min_* on day 161 and NDVI*_max_* on day 129. While the NDVI ratio of the SOS and EOS for maize was determined according to NDVI*_min_* on day 161 and NDVI*_max_* on day 241, and NDVI*_min_* on day 281 and NDVI*_max_* on day 241, respectively. Later, the threshold values were calculated based on the highest probability of NDVI ratio within the study area. Finally, crop phenological features, including the SOS and the EOS, were extracted based on the thresholds and the NDVI time series in the TIMESAT software [36], that is if the NDVI values reached the thresholds of SOS and EOS for pixels in the images, we considered it’s the time of SOS and EOS.

### 3.4. Accuracy Assessment

Three statistical criteria, the coefficient of determination (*R*^2^), the mean absolute error (MAE) and the root mean square error (RMSE) were selected to evaluate the estimated SOS and EOS. Due to the small amount of observational phenology data (15 for winter wheat and 18 for summer maize), it was quite inefficient to withhold part of the data to evaluate the phenology extraction results; therefore, the leave-one-out cross-validation (LOOCV) approach was selected to examine the accuracy of the estimation models [37]. The LOOCV method involves using one observation as the validation sample and the remaining observations as the training samples; this procedure was repeated *N* (number of the observation values) times. The *R*^2^, MAE and RMSE of the algorithm were then estimated through averaging the values obtained from the *N* iterations:

(3) $$R^{2}=1-\frac{\sum_{i=1}^{n}{\left(M_{i}-F_{i}\right)}^{2}}{\sum_{i=1}^{n}{\left(M_{i}-A_{i}\right)}^{2}}$$

(4) $$\mathrm{MAE}=\frac{1}{n}\times \sum_{i=1}^{n}\left|M_{i}-E_{i}\right|$$

(5) $$\mathrm{RMSE}=\;\sqrt{\frac{1}{n}\overset{n}{\underset{i=1}{\sum }}{\left(M_{i}-E_{i}\right)}^{2}}$$

where *n* is the number of observations, $M_{i}$ is the measured value, $F_{i}$ is the linear fitting value, $A_{i}$ is the average value of the measured data and $E_{i}$ is the estimated value.

## 4. Results

### 4.1. The STARFM Prediction Results

**Figure 4:**
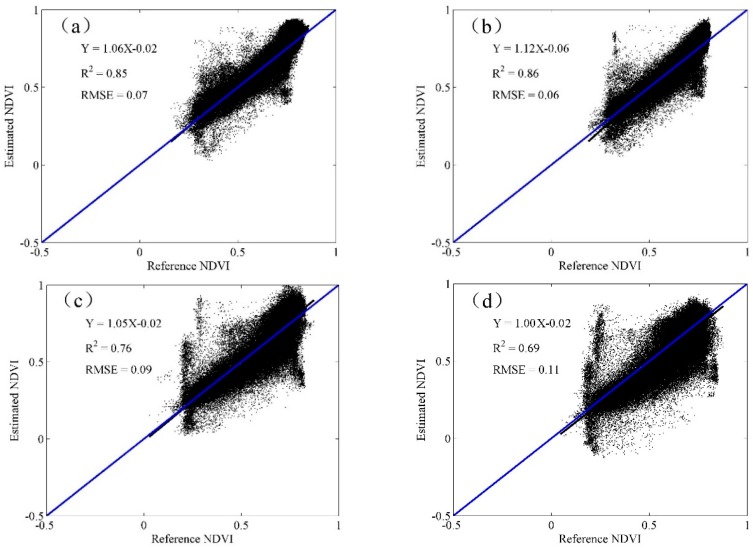
Relationships between the blended and observed data for the wheat season (**a**) DOY121; (**b**) DOY129; (**c**) DOY137; and (**d**) DOY145.

**Figure 5:**
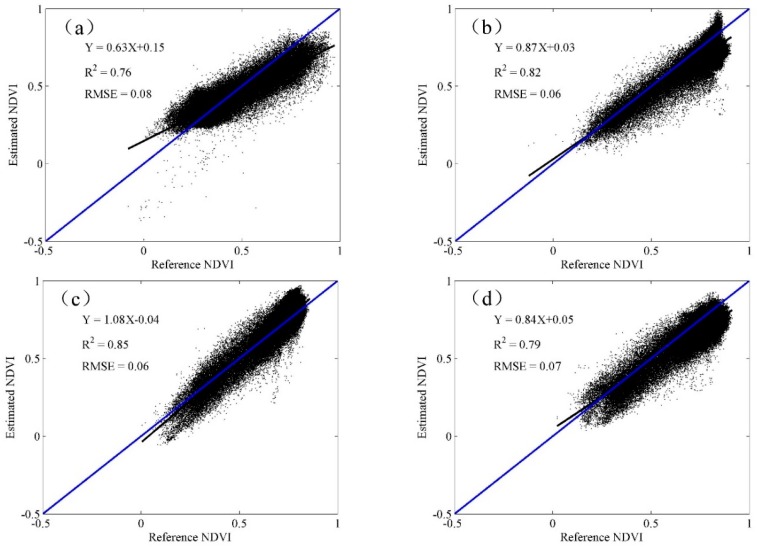
Relationships between the blended and observed data for the maize season (**a**) DOY177; (**b**) DOY217; (**c**) DOY233; and (**d**) DOY257.

Comparisons between the predicted and observed NDVI are provided in Figure 4 and Figure 5. The scatterplots show that all the predicted data were closer to the 1–1 line, which demonstrates the good performance of STARFM algorithm in the fusion of SPOT5 and MODIS products. The *R*^2^ and RMSE were chosen to measure the strength of the relationship between the estimated results and the reference data. Overall, the *R*^2^ was higher than 0.69, and the RMSE was lower than 0.11 for all predicted data. Specifically, the *R*^2^ was between 0.69 and 0.86, and the RMSE was between 0.06 and 0.11 in the wheat season. Meanwhile, the *R*^2^ ranged from 0.76 to 0.85, and the RMSE ranged from 0.06 to 0.08 between the predicted and observed NDVI values during the maize season.

The results also show that correlations between the blended and referenced NDVI were lower for DOY137 than for DOY121 and DOY129 in the wheat season, and the correlations were lower for DOY257 than for DOY233 during the maize season. This indicated that the accuracies of the predicted data decreased when the time interval increased, a finding consistent with several previous studies [23], which may be due to the probability of land surface and sun zenith angle changes that increase with increasing time spans. The prediction accuracy was significantly decreased for DOY145 and DOY177. This is most likely caused by intense farming activities such as harvesting and sowing occurring at the end and start of the growing season of the two crops, respectively.

**Figure 6:**
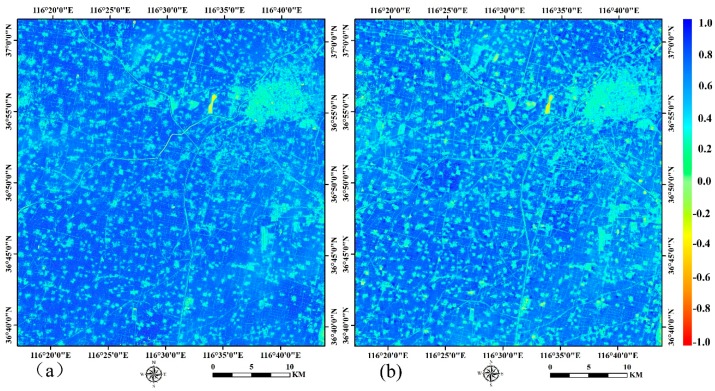

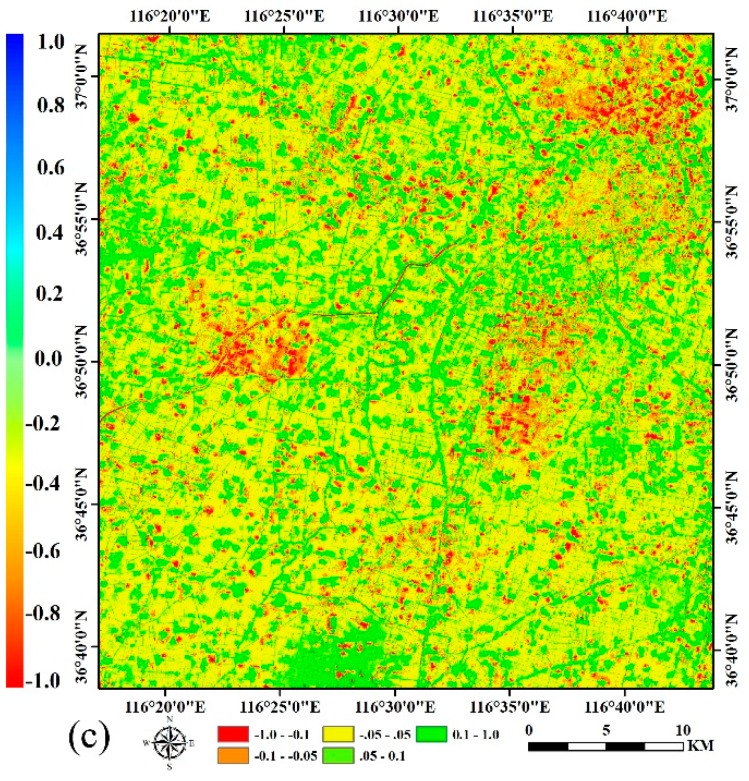
The comparison of predicted and reference NDVI of DOY137. (**a**) The reference SPOT5 NDVI (**b**) The predicted NDVI; (**c**) The difference of predicted NDVI in (**a**) and reference NDVI in (**b**).

Figure 6 shows the visual comparison of predicted and reference NDVI, from which we can obtain that they had good consistency because most of the difference values presented in Figure 6c were between −0.10 and 0.10. In the following research, all ten SPOT5 images were used to predict high spatio-temporal resolution NDVI, and only the nearest SPOT5 images in both the forward and backward temporal directions were used for prediction in order to rely on high-resolution observations that were as close as possible to the prediction dates. This indicated that the NDVI prediction accuracy may be higher than the accuracy given above. Additionally, the real SPOT5- and the STARFM-predicted images were combined when detecting the crop phenology parameters.

### 4.2. Crop Classification and Mapping

**Figure 7:**
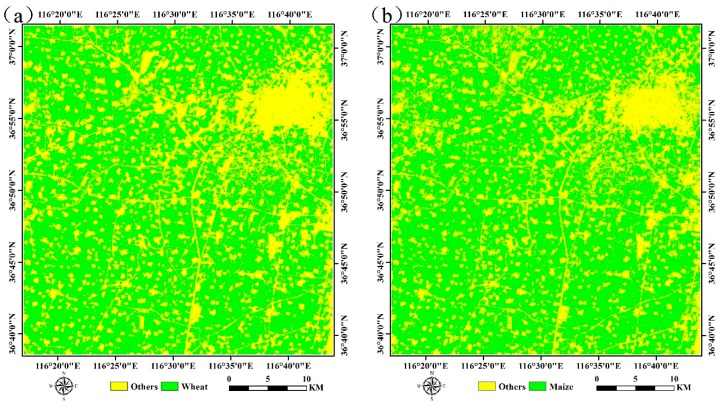
Classification results of different crops using SPOT5 images (**a**) for winter wheat; (**b**) for summer maize.

Based on the available multi-temporal SPOT5 images and SVM method, the distribution maps of wheat and maize were generated (Figure 7). As shown in the two figures, most of the wheat and maize fields were overlapped, indicating that the planting area kept stable. The classification results were evaluated using the ground survey data (92 points for wheat and 104 points for the other types during the wheat season; and 103 points for maize and 123 points for the other types during the maize season), and the assessment indicators contains producer’s accuracy, user’s accuracy, overall accuracy and kappa coefficient.

Table 3 and Table 4 provide the results of the assessment, respectively, from which we can obtain that the overall accuracies for both wheat and maize were above 86%, and the kappa coefficients were higher than 0.73 when using SPOT5 images, which is acceptable for crop mapping. Meanwhile, the overall accuracies for both wheat and maize were below 75%, and the kappa coefficients were lower than 0.50 when involved the MODIS data, which demonstrated that the classification accuracies were improved with the spatial resolution increasing. This mainly because it’s easier to produce mixed pixels in the coarse or moderate spatial resolution images and thus result in the relatively low classification accuracies.

### 4.3. Crop Phenology Extraction and Mapping

**Figure 8:**
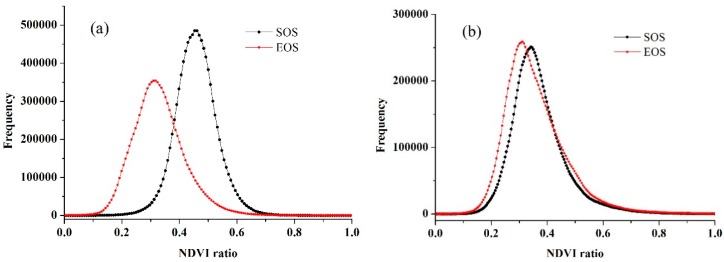
Histogram distributions of the NDVI ratio based on the blended data (**a**) for wheat and (**b**) for maize.

**Figure 9:**
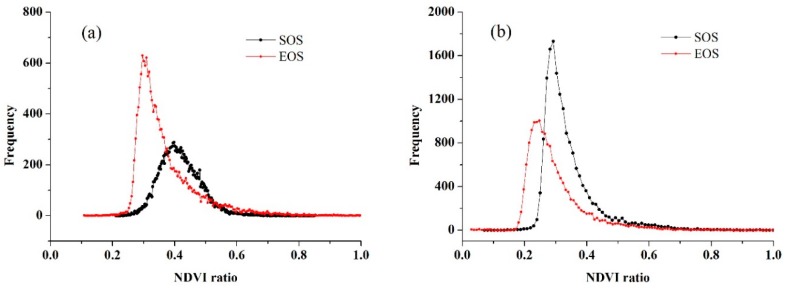
Histogram distributions of the NDVI ratio based on MODIS data (**a**) for wheat and (**b**) for maize.

Based on the smoothed data using the S-G filtering, the thresholds of the SOS and EOS were determined. Figure 8 shows the histograms of the NDVI ratio of wheat and maize, all of which were similar to normal distributions. According to the highest probability of the frequencies of NDVI ratio, the thresholds of the SOS and EOS for wheat were 0.45 and 0.32, and the thresholds of the SOS and EOS for maize were 0.35 and 0.31, respectively. To compare the crop phenology estimation accuracy at different spatial resolutions, we extracted the SOS and EOS of wheat and maize in both the time-series MODIS data at the 250 m resolution and the predicted data at the 10 m resolution with the same selected threshold determination methods. Figure 9 shows the frequency distributions of the NDVI ratio of wheat and maize based on the time-series MODIS NDVI, where the thresholds of the SOS and EOS for wheat were 0.40 and 0.30 and the thresholds of the SOS and EOS for maize were 0.29 and 0.25. Overall, the threshold of the SOS and the EOS for wheat was larger than that for maize, and the threshold of the SOS was larger than that of the EOS for each individual crop.

**Figure 10:**
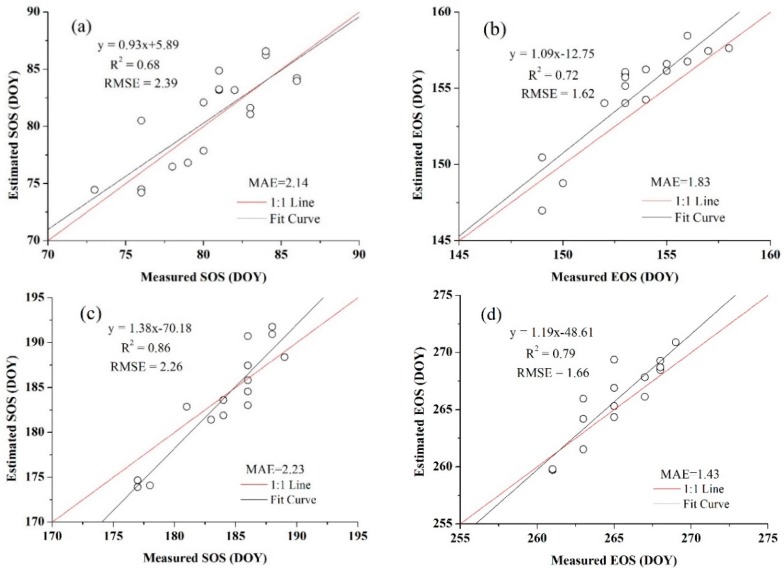
Comparisons of the field-measured phenology with the predicted phenology using blended NDVI (**a**) for the SOS of wheat; (**b**) for the EOS of wheat; (**c**) for the SOS of maize; and (**d**) for the EOS of maize.

**Figure 11:**
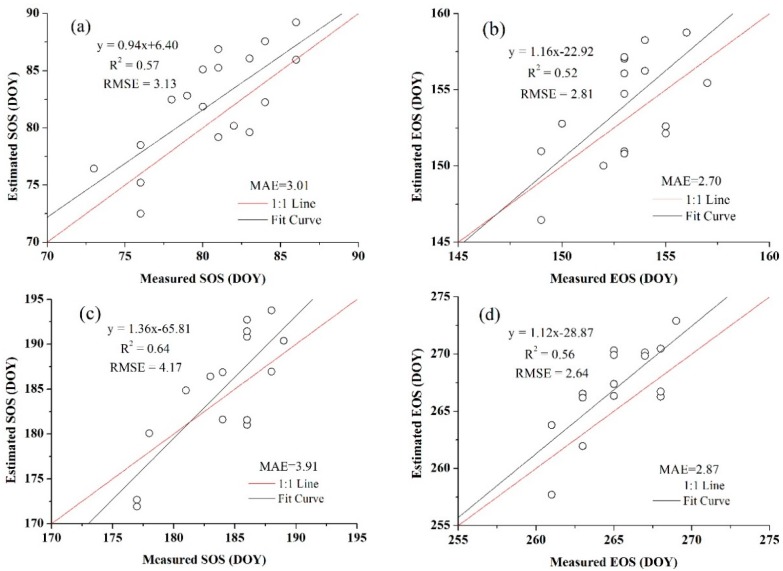
Comparisons of the field-measured phenology with the predicted phenology using MODIS NDVI (**a**) for the SOS of wheat; (**b**) for the EOS of wheat; (**c**) for the SOS of maize; and (**d**) for the EOS of maize.

**Figure 12:**
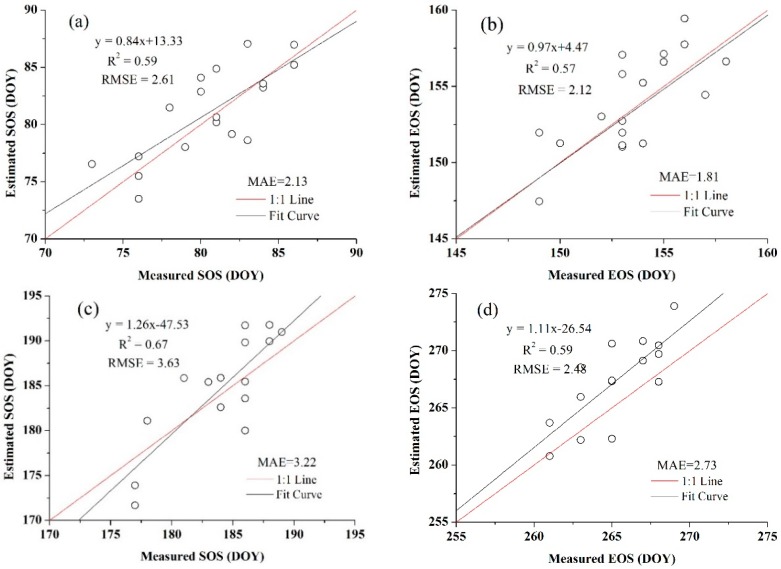
Comparisons of the field-measured phenology with the predicted phenology using the blended NDVI which have aggregated to MODIS’s resolution (**a**) for the SOS of wheat; (**b**) for the EOS of wheat; (**c**) for the SOS of maize; and (**d**) for the EOS of maize.

**Figure 13:**
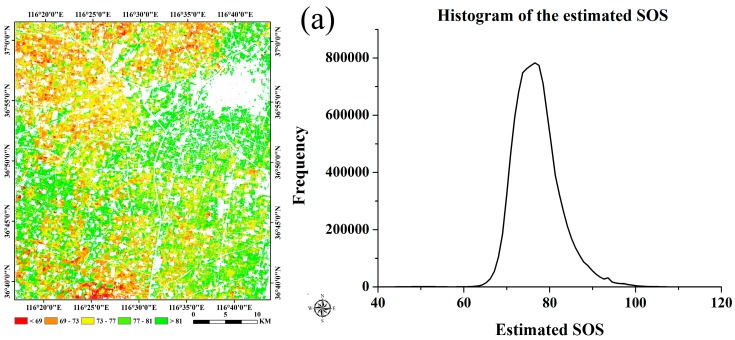

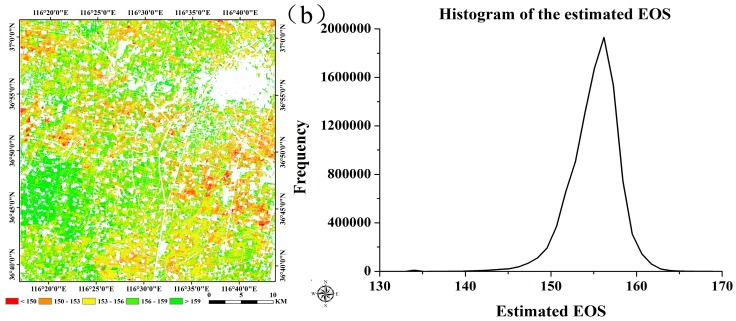
Maps and histograms of the winter wheat phenology (**a**) for the SOS and (**b**) for the EOS.

**Figure 14:**
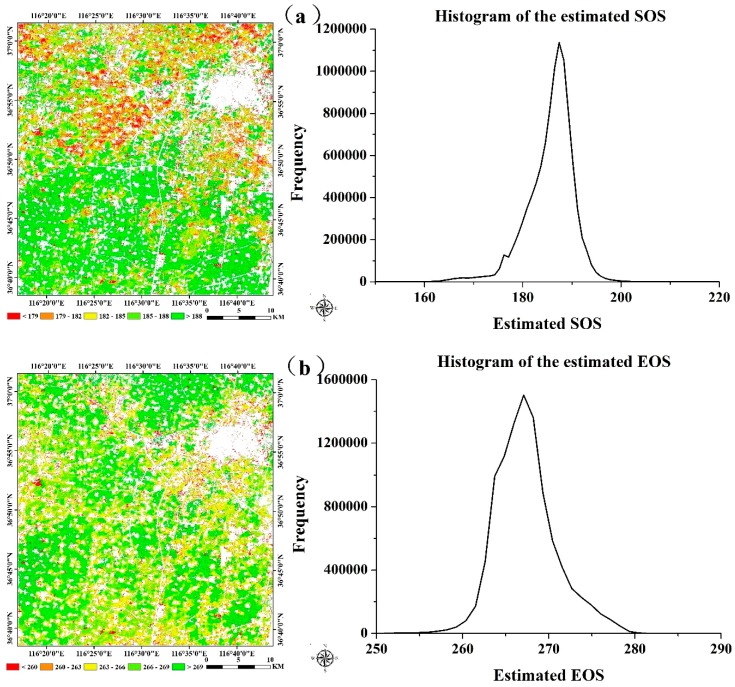
Maps and histograms of the summer maize phenology (**a**) for the SOS and (**b**) for the EOS.

In addition, the threshold of SOS and EOS for both crops obtained from the blended data were more representative than the threshold that achieved from the MODIS products because they were more consistent with the normal distribution, which also showed the benefits of using high spatio-temporal resolution data. The relationships between the estimated SOS and EOS dates and the observation data are presented in Figure 10 and Figure 11. Figure 10 is the extraction result based on the time-series blended NDVI (10 m resolution), and Figure 11 shows the extraction result using the time-series MODIS NDVI (250 m resolution). As shown in Figure 10, all the estimations were closely related to the observations, with an *R*^2^ greater than 0.67 and an RMSE lower than 2.40 days, and an MAE of less than 2.30 days. For the SOS of maize, the prediction obtained the highest *R*^2^ (0.86) and a relatively low RMSE (2.26 days), whereas the smallest *R*^2^ (0.68) and largest RMSE (2.39 days) can be observed in the SOS estimation for wheat. Although the *R*^2^ of the SOS and EOS was 0.57 and 0.52 for wheat, it was 0.64 and 0.56 for maize when using the MODIS NDVI (Figure 11). Overall, the accuracies of estimations based on the time-series blended data with higher *R*^2^, lower RMSE and MAE were better than the results using MODIS data, which was also mainly due to the influence of mixed pixels. To minimise the influence of differences in spatial resolution in accuracy evaluation and demonstrate the practical effect of applying this generated NDVI dataset in crops phenology extraction, the blended NDVI was aggregated to the resolution of MODIS and then extracted phenology to spatially match the estimation result derived from the original MODIS data. Improvement in accuracy can be seen from the comparison between Figure 11 and Figure 12, the *R*^2^ between estimated and observed phenology has increased, while the RMSE and MAE has decreased. All of this have indicated the feasibility of the proposed framework for crop phenology monitoring. Based on the methodologies described above and the spatial distributions of the two crops, the maps and histograms of phenology parameters using the blended time-series NDVI were generated and are presented in Figure 12 and Figure 13. As shown in the four figures, the histograms of the SOS and EOS also appear somewhat similar to normal distributions, and most of the SOS and EOS values were between DOY70–DOY85 and DOY150–DOY165 for wheat and between DOY179–DOY195 and DOY260–DOY275 for maize, indicating that the maximum difference of the SOS and EOS within the study area was approximately half a month for both wheat and maize. This demonstrated estimated results were acceptable within a specific range, and therefore the spatial distributions of crop phenology can provide favourable supplement for the observation from agro-meteorological station. Moreover, there is little overlap between estimated EOS in Figure 13 and estimated SOS in Figure 14, the most likely reason is that the farmland belonged to many different farmers in China and lacked unified management, so when to sow, fertilize and harvest was decided by each one, and therefore led to this phenomenon.

## 5. Discussion

Since numerous studies on vegetation phenology detection have been conducted using coarse-resolution data, such as the AVHRR, MODIS, and SPOT-VGT time series at regional scales and few studies were found using high-spatial-resolution data for crop phenology monitoring at small-field scales, we proposed a framework for phenology detection using high spatio-temporal resolution data blended from SPOT5 and MODIS products in this paper. The research provided an approach for crops phenology detection at a finer scale and achieved favourable results, and also it showed various advantages of applying the time-series fused high spatial resolution data than the coarse spatial resolution data for phenology monitoring in the study region. Therefore, our study is very meaningful for the implementation of precision agriculture. However, several problems listed as below necessitate further improvements though we were able to accurately extract phenology.

### 5.1. High Spatial Resolution Data

**Figure 15:**
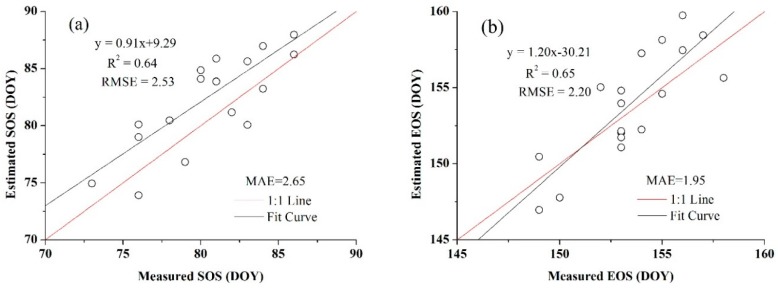

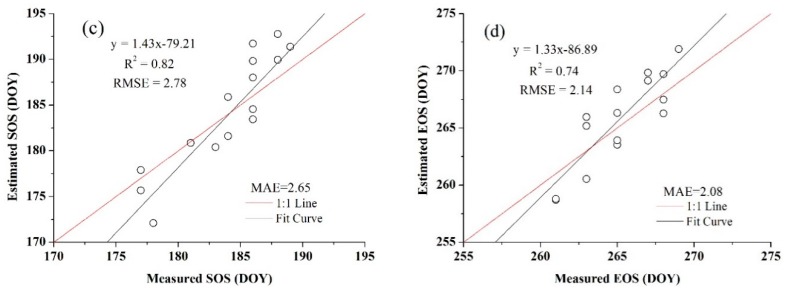
Comparisons of the field-measured phenology with the predicted phenology using time-series NDVI blended from Landsat8 and MODIS (**a**) for the SOS of wheat; (**b**) for the EOS of wheat; (**c**) for the SOS of maize; and (**d**) for the EOS of maize.

Besides the limitation that we used the 8-day MODIS data instead of the daily data, which may led to low estimation accuracy, the major limitation is that an insufficient number of SPOT5 images were available at the early stages of wheat, which may thus cause inaccurate predictions because of the long-time spans between the input data pair and the predicted date. In our study, the accuracies of synthetic data in the early season were still reliable, although they may not be as accurate as the predictions that were close to the input SPOT5 data because the land surface of the planted areas remained stable, as most agricultural activities were suspended during this period. For the implementation of STARFM algorithm, even if only one base high-spatial-resolution image exists it also could predict time-series high-spatial-resolution data covering the whole growing season of crops, greater access to high-spatial-resolution data may result in higher fusion accuracies and hence improve the performance of crop phenology identification. In this study, the strong potential of fusing high-spatial-resolution SPOT5 and high-temporal-resolution MODIS data for phenology detection has been proven, but since the SPOT5 images are acquired from commercial satellite which need to buy, we would like to test the free source and widely used data obtained from Landsat to extract phenology parameters, Figure 15 presented relationships between the phenology estimation results using high spatio-temporal resolution data blended from two image pair of Landsat8 OLI and MODIS and the field observations, from which we can see although the correlations (*R*^2^ were between 0.64 and 0.82, RMSE were between 2.14 and 2.78 days, and MAE were between 1.95 and 2.65 days) were lower than the estimations involving SPOT5 data, they were significantly correlated with the measured values. This demonstrated the feasibility of utilizing Landsat data to detect crops phenology at high spatial resolution when the commercial SPOT5 images are unavailable. Exhilaratingly, with recent satellite missions, such as Europe’s Sentinel-2 and Sentinel-3 and China’s Gaofen series, new possibilities emerge for data fusion approaches extending to products from other space-borne sensors, which will contribute to better crop phenology monitoring.

### 5.2. Smoothing Methods

The smoothing algorithm is also an important limitation that may influence phenology extraction from satellite images. In this study, we tested three filtering methods in the TIMESAT software, and we used the RMSE to quantitative analysis the difference between the observed and smoothed values. The RMSE values are presented in Table 5, from which we can determine that S-G performed better among the three filtering methods as it had lowest RMSE, whether for wheat (0.0152) or for maize (0.0272). Therefore, the S-G filter is considered more suitable for the time-series NDVI reconstruction. However, numerous other smoothing techniques have been developed for processing time-series remote sensing data, and no agreement has been reached on which filter performs best [38]. As this is preliminary and exploratory research, we did not discuss substantially more issues about which filter is suitable or better; this is beyond the scope of this study. Further research should make additional comparisons of using different filtering methods to estimate phenology parameters. In addition, unfavourable weather conditions, such as cloud cover, are another restriction for phenology monitoring. Although the MVC technique and smooth filtering can attenuate the influence of intermittent cloud cover, the presented method may not perform well in regions with successive cloud covers, which will lead to large data gaps in the NDVI time series and ultimately affect the accuracy of the extraction phenology.

Here, the RMSE value indicate the difference between the mean NDVI time series obtained from the three noise-reduction techniques and the corresponding experimental time series to which noise reduction has been applied. The smaller the value, the better of the filter.

### 5.3. Data Fusion Algorithms

Because the phenology estimations are mainly based on time-series analysis when using remote sensing techniques, the reliability and stability of data fusion methods are very important because they may greatly influence the accuracy of the blended high-spatial-resolution data. In this paper, we only explored the potential of the STARFM algorithm for fusing SPOT5 and MODIS products. Although its capability has been demonstrated, the prediction accuracy is also affected by limitations of the fusion algorithm. For example, even though the fields are very small in our study area, some of them are adjacent with the same crops. In a way they behave homogenously, a bit like mega-fields (composed of many small fields), so it may be easier to produce high spatial resolution data. But inaccurate prediction results may occur in planting regions with complex crop types across adjacent small-scale fields, such as Austria and Sahelian region in Europe and Africa, respectively [39,40]. Moreover, the STARFM algorithm cannot predict change events if the disturbances are not recorded in at least one of the base high-spatial-resolution images [15,17]. Fortunately, a number of researchers are devoted to improving the prediction accuracies and extending applications of the fusion techniques. For example, the STAARCH and ESTARFM algorithms were proposed in an attempt to overcome several limitations of the STARFM algorithm [14,17]. Recently, a framework for the ESTARFM algorithm was developed to make it applicable for large, cloud-prone and heterogeneous areas [41]. For future improvements, we will thoroughly test the existing data fusion methods and appraise their applications in phenology detection.

### 5.4. Geometric Accuracy and PSF

The geometric accuracy and point spread function (PSF) may also influence the results of phenology estimations. Since we used several pairs of SPOT5 and MODIS images to predict desired high spatial resolution data, if there are geometric errors in these images, they will mismatched and the predicted high spatial resolution data will not be so accurate. Moreover, the geometric accuracy also can influence the validation of the phenology extractions. The PSF of a sensor describes how much of a signal reaching a detector element actually comes from adjacent areas outside the nominal observation areas of the pixel and it weighs the signal over the image plane contributing to the detector readout [42,43]. Therefore, when computing vegetation index for each low spatial resolution pixel, PSF effects should be taken into account and the corresponding weights should be used to compute the fractional coverage of each land use types [44]. According to Schowengerdt, the sensor PSF which has been modelled in several studies includes several components: the optical PSF, the image motion PSF, the electronic PSF, and the detector PSF [43,44,45,46]. Even though for some remotely sensed images, such as the MERIS, the PSF can be negligible for the unmixing-based data fusion approach [44,47], it should be noted that this is not the case when using MODIS data because its triangular PSF results in overlap between adjacent observations such that 25% of the signal is from adjacent areas [48]. Therefore, the MODIS PSF also should be taken into account for the improvements of data fusion algorithms and phenology detections in the future’s research.

### 5.5. VIs’ Selection and Influence

Finally, since many VIs can be derived from hyperspectral images and since some of them are widely used in the estimation of vegetation biochemical and biophysical variables [49,50,51], further studies should be focused on assessing the performance of different VIs in phenology monitoring when using remotely sensed time-series data. Recently, several studies have achieved some progress in this interesting field. For example, Meng et al. found that EVI (Enhanced Vegetation Index) and NDWI (Normalized Difference Water Index) performed better than SAVI (Soil Adjusted Vegetation Index) and NDVI in the optimal harvest date extraction for soybean [52]. The WDRVI (Wide Dynamic Range Vegetation Index) was selected by Sakamoto et al. to detect maize and soybean phenology because this index has been proven to have a higher sensitivity to changes at moderate to high biomass compared to the NDVI [5]. In the future, we should like to assess the performance of other VIs (e.g., EVI and WDRVI) and evaluate their suitability and capability for crop phenology estimation.

## 6. Conclusions

The detection of crop phenology at a high spatial resolution is of crucial importance for agriculture management in China, which is a large agricultural country with high fragmented fields. In this study, we proposed a framework to map crop phenology using high spatio-temporal resolution data blended from the SPOT5 and MODIS products and verified its feasibility in an agricultural county in Shandong Province, China.

Our results show that: (1) the STARFM algorithm possesses the ability to blend SPOT5 and MODIS data. The *R*^2^ between the predicted and observed NDVI ranged from 0.69 to 0.86 and from 0.76 to 0.86 during the wheat season and maize season, with acceptable RMSEs; In addition, (2) the fused datasets have the potential to detect crop phenology at a high spatial resolution. Based on the threshold method, the SOS and EOS that were extracted from the time-series NDVI were consistent with the field-observed data, with the *R*^2^ of the SOS varying from 0.68 to 0.86 and the *R*^2^ of the EOS varying from 0.72 to 0.79; Finally, (3) the accuracy of phenology estimations that involved blended data was better than that using MODIS data, indicating the strong feasibility and reliability of the proposed framework for phenology monitoring.

This research demonstrates the potential of using high spatial and temporal resolution data blended from the STARFM algorithm to detect crop phenology. Here, it was only used to fuse SPOT5 and MODIS data, but it is not limited to these two products. The proposed approach can easily be used with other satellite images for accurate phenology monitoring at finer scales.

## Figures and Tables

**Table 1:** Phenology stages and exact date in the study area. SOS: start of season; EOS: end of season; DOY: day of the year.

Winter Wheat		Summer Maize	
Phenology Stage	Date (DOY)	Phenology Stage	Date (DOY)
Sowing	288	Sowing	163
Emergence	298	Emergence	169
Tillering	325	Seven leaf	186 (SOS)
Wintering	349–51 (next year)	Tasseling	222
Jointing	81 (SOS)	Silking	227
Booting	103	Maturity	265 (EOS)
Maturity	153 (EOS)	Harvest	274
Harvest	160	-	-

Note: the date of each phenology in this table is the average value of date from all the sampling sites.

**Table 2:** SPOT5 image acquisitions used in this study.

Wheat Season		Maize Season	
Acquisition Date (Month/Day)	DOY	Acquisition Date (Month/Day)	DOY
4/23	113	7/2	183
5/8	128	8/11	223
5/13	133	8/16	228
5/23	143	8/21	233
5/28	148	9/15	258

**Table 3:** Classification accuracies of winter wheat using the SPOT5 and MODIS data.

Class	SPOT5 Data		MODIS Data	
	Producer’s Accuracy	User’s Accuracy	Producer’s Accuracy	User’s Accuracy
Wheat	89.13%	83.67%	73.91%	67.33%
Others	84.62%	89.79%	68.26%	74.74%
	Overall Accuracy: 86.73%; Kappa: 0.7347		Overall Accuracy: 70.92%; Kappa: 0.4210	

**Table 4:** Classification accuracies of summer maize using the SPOT5 and MODIS data.

Class	SPOT5 Data		MODIS Data	
	Producer’s Accuracy	User’s Accuracy	Producer’s Accuracy	User’s Accuracy
Maize	91.26%	87.85%	75.73%	70.91%
Others	89.43%	92.44%	73.98%	78.45%
	Overall Accuracy: 90.27%; Kappa: 0.8044		Overall Accuracy: 74.78%; Kappa: 0.4944	

**Table 5:** RMSE values for the three techniques of data filtering.

Filtering Methods	Crop Types	
	Winter Wheat	Summer Maize
A-G	0.0158	0.0291
D-L	0.0235	0.0453
S-G	0.0152	0.0266
